# Simultaneous Isolation of Three Different Stem Cell Populations from Murine Skin

**DOI:** 10.1371/journal.pone.0140143

**Published:** 2015-10-13

**Authors:** Maria Fernanda Forni, Aline Ramos Maia Lobba, Alexandre Hamilton Pereira Ferreira, Mari Cleide Sogayar

**Affiliations:** 1 Núcleo de Terapia Celular e Molecular (NUCEL/NETCEM), Departamento de Clínica Médica, Faculdade de Medicina, Universidade de São Paulo, São Paulo, 05360-130 SP, Brasil; 2 Instituto de Química, Departamento de Bioquímica, Universidade de São Paulo, São Paulo, 05508-000 SP, Brasil; University of Maryland School of Medicine, UNITED STATES

## Abstract

The skin is a rich source of readily accessible stem cells. The level of plasticity afforded by these cells is becoming increasingly important as the potential of stem cells in Cell Therapy and Regenerative Medicine continues to be explored. Several protocols described single type stem cell isolation from skin; however, none of them afforded simultaneous isolation of more than one population. Herein, we describe the simultaneous isolation and characterization of three stem cell populations from the dermis and epidermis of murine skin, namely Epidermal Stem Cells (EpiSCs), Skin-derived Precursors (SKPs) and Mesenchymal Stem Cells (MSCs). The simultaneous isolation was possible through a simple protocol based on culture selection techniques. These cell populations are shown to be capable of generating chondrocytes, adipocytes, osteocytes, terminally differentiated keratinocytes, neurons and glia, rendering this protocol suitable for the isolation of cells for tissue replenishment and cell based therapies. The advantages of this procedure are far-reaching since the skin is not only the largest organ in the body, but also provides an easily accessible source of stem cells for autologous graft.

## Introduction

The skin is the primary barrier that protects the body from dehydration, mechanical trauma, and microbial insults, consisting of an outermost epidermis and appendages, being separated from the underlying dermis by a basement membrane [1]. Continuously undergoing self-renewal to repair damaged tissue and replace old cells, this tissue depends on stem cell populations, which reside in the adult hair follicle, sebaceous gland, dermis and epidermis [2]. Interfollicular epidermal stem cells, named EpiSCs hereforth, rely on an underlying basement membrane which is rich in extracellular matrix proteins and growth factors. Basal cells attach to this structure through adhesion complexes such as hemidesmossomes containing a core of α6β4 integrins and focal adhesions of α3β1 integrins. These proteins also play a role in growth control and migration [3]. The α6 and β1 integrins were taken as markers of epidermal stem cells [Reviewed in [4–6], as well as p63, a p53 homologue that is expressed through the basal layer of the epidermis [7] with a putative function in maintaining these cells in a slow cycling state. These epidermal stem cells are responsible for a rapidly dividing progeny referred to as transit amplifying, which undergoes a limited number of divisions before withdrawing from the cell cycle, commiting to terminal differentiation, and migrating towards the surface of the skin, generating dead, flattened, differentiated keratinocytes [8]. The intermediate filaments filaggrin and involucrin are expressed during this process, being specific markers of epidermal differentiation [8]. These cells were first described by Jones and cols in 1995 [9] and several enrichment protocols have been reported in the literature for the isolation of EpiSCs, based on β1 integrin expression [3], α6 and CD71 [10] or Hoescht 33342 exclusion, combined with cell size [11]. In 2001, Toma and cols described a multi-potent precursor cell population from adult mammalian dermis, more specifically, in the follicle dermal papillae [12]. These cells, termed SKPs, for skin-derived precursors, were isolated and expanded from rodent and human skin and differentiated into both neural and mesodermal progeny, including cell types never found in the skin, such as neurons. These cells expressed markers of neuronal precursors, such as Nestin, and mesenchymal cell lines such as Vimentin, but not Fibronectin. Later on, the same group proposed that SKPs represent a multi-potent neural-crest-like precursor that arises in embryonic mammalian tissues, and is maintained throughout adulthood [13]. This may explain why SKPs are capable of differentiation into βIII tubulin, CNPase, and GFAP neural precursors. In vivo, these cells were capable of generating myelinating Schwann cells, a fact of great impact in the area of spinal cord injury treatment [14,15]. The mesenchymal skin stem cells reside in the bulge of hair follicles. Expression of CD34 as a marker for mouse bulge stem cells was first described by Trempus et al [16]. Interestingly, CD34 is a hematopoietic stem cell marker in human bone marrow, but not in the mouse and is not expressed in the human bulge region. Thus, CD34 cannot be used for human hair follicle bulge cell isolation. However, at present, CD34 represents the best marker for mouse hair follicle bulge cells, providing a valuable tool for studying bulge cell biology. Recently, a CD34^+^ mesenchymal cell population of murine skin was isolated [17] and proven to be capable of osteogenesis, chondrogenesis and adipogenesis *in vitro*. In brief, several protocols attempted to isolate different stem cell populations from the skin [7,10–14,16,17], but none of them allowed the simultaneous isolation of more than one SC population [18]. In future attempts to use skin as a source of stem cells for cell therapy, the variety of differentiated cell types would be of great importance. A possible solution for this conflicting scenario would be the simultaneous isolation of more than one population of skin stem cells. Stimulated by these remarkable facts, herein we describe a simple method, based on enzyme treatments and culture selection techniques, which allows the isolation of three different stem cell populations, with different potentiality and plasticity, from murine dermis and epidermis. Our findings also encourage the future possibility to translate this technique to human skin stem cells isolation.

## Materials and Methods

### Animal handling, Euthanasia and Tissue collection

Euthanasia of two to three months old male Balb/c mice was performed by introduction of 100% carbon dioxide into a bedding-free cage initially containing room air with the lid closed at a rate sufficient (fill rate of 25% of the chamber volume per minute) to induce rapid unconsciousness with minimal distress to the animals. Once euthanasia was performed, collection of tissues was initiated immediately. All the procedures were in agreement with the University of São Paulo Chemistry Institute Ethical Committee guidelines. The protocol was approved by the Ethics Committee for Animal Experimentation of the University of São Paulo (Permit Number: 2007/02).

### Isolation of Skin Stem Cells

Back skin scalps were shaved and the skin was cut into 2 to 3 mm^2^ fragments and exposed to Liberase (catalog number # 5401160001, 1mg/ml, Roche, Germany) in HBSS (Hanks Balanced Salt Solution) for 1h at 37°C. After mechanical dissociation, the cells obtained were incubated in DMEM (Gibco, Rockville, MD) supplemented with 10% fetal bovine serum (FBS), centrifuged and ressuspended in SKP and mesenchymal culture media. The resulting tissue (comprising the epidermis) was exposed to Dispase (catalog number #17105–041, Life Technologies, USA) 100-caseinolytic units/mL for 30min at 37°C. Following mechanical dissociation, the cells were plated in EpiSC medium in collagen IV coated plates. After 24h, the adherent cultures were exposed to 0.1% trypsin for 2min, the floating cells were discarded and fresh medium was added. The first passage was carried out one week later. All the adherent cells were subcultured everytime that they reached 70% confluency (in general twice a week), always respecting the dilution ratio of 1:3 of the plate area during the consecutive passages. For the floating cell clusters subculturing was performed weekly.

### Cell Culture

Epidermal Stem Cells were sub-cultured in KSFM (Gibco, Rockville, MD) supplemented with 20ng/ml EGF, EPB and 1% ampicilin/streptomycin in collagen IV coated plates (catalog number # 3410-010-01, R&D Systems, USA). The mesenchymal stem cells were maintained in DMEM (Gibco, Rockville, MD) containing 10% FBS and 1% ampicilin/streptomycin in adherent flasks. Skin precursors were kept in DMEM: F12 at a 3:1 ratio (Gibco) with 20ng/ml EGF (catalog number # 236-EG-01M, R&D Systems, USA), 40ng/ml bFGF (catalog number # 233-FB-01M, R&D Systems, USA), 1% B27 supplement (catalog number # 17504044, Invitrogen, USA), The cells were maintained at 37°C, 2% CO_2_ and controlled humidity.

### Differentiation Assays

EpiSCs differentiation was induced with SMEM (Gibco, Rockville, MD, USA) containing 0.05mM Ca^2+^, 10% FBS, 1% ampicilin/streptomycin in non-coated plates as described in [3]. SKPs differentiation was induced with DMEM:F12 at a ratio of 3:1 (Gibco, Rockville, MD), 15% fetal bovine serum (FBS), in poly-L-lysine coated plates (catalog number # P7280, Sigma, USA) as described in [12,13]. The mesenchymal cells were induced to adipogenesis with DMEM-high glucose (Gibco, Rockville, MD), 10%FBS, 1uM dexamethasone (catalog number # D4902-25MG, Sigma, USA), 0.5mM IBMX (catalog number # I7018-250MG, Sigma, USA), 10ug/ul Insulin (catalog number # I1882-100MG, Invitrogen, USA), 100uM indomethacin (catalog number # I7378-5G, Sigma, USA). Chondrogenesis: DMEM-F12 (Gibco, Rockville, MD) 3:1, 0.5ug/ml Insulin (catalog number # I1882-100MG, Invitrogen, USA), 50uM ascorbic acid (catalog number # A4544-25G, Sigma, USA), 10ng/mL TGFβ-1 (catalog number # 7666-MB-005, R&D Systems, USA) and 1mM sodium piruvate (catalog number # 11360–070, Sigma, USA). Osteogenesis: αMEM (Gibco, Rockville, MD), 10% FBS, 0.1uM dexamethasone (catalog number # D4902-25MG, Sigma, USA), 2mM ascorbic acid (catalog number # A4544-25G, Sigma, USA), and 10mM Glycerol 2-Phosphate (catalog number # G9422-10G, Sigma, USA) as described in [19].

### Immunocytochemistry and Immunofluorescence

Attached cells were enzymatically dissociated into single cells before plating onto glass slides coated with poly-L-lysine (Sigma, USA). Floating SKP spheres were laid down on the slides through cytospin centrifugation (5min, 1,000 rpm). The medium was changed every 3 to 4 days. Cells were fixed with 4% paraformaldehyde and after blocking and permeabilizing with 2% BSA and 0.1% Triton X-100 in PBSA (PBS without calcium or magnesium), the slides were incubated with primary antibodies in 2% BSA at 40°C, overnight. Primary antibody binding was detected through the corresponding secondary antibodies. Between each immunoglobulin exposure, cells were washed three times with PBSA, at room temperature, for 5 min. Cells were counterstained with DAPI (Sigma, USA) to display the nuclei. The slides were observed under a Zeiss LSM 510 Confocal Microscope (Carl Zeiss Inc, Thornwood, NY, USA). Positive and negative controls may be observed in the Supplementary Figure (S2 Fig). Osteogenic differentiation detection was determined using the Alizarin Red S Staining protocol, adipogenic differentiation with Oil Red O and chondrogenesis with Toluidine Blue staining, as described elsewhere [14].

### RNA Isolation and Reverse Transcriptase Real-Time PCR

Total RNA was isolated using the RNAEasy kit (GE Healthcare). RNA quality control was assessed through the 280/260nm and 230/260nm ratio (> 1.8 was taken as adequate purity) and by observing the integrity of the 18S/28S bands in a denaturing gel. The cDNA was obtained using Super Script III (Invitrogen, Carlsbad, CA). Amplification of the resulting cDNAs was achieved using the SYBR GREEN Dye (Applied Biosystems) in a GeneAmp 7500 Sequence Detection System (Applied Biosystems) under the following conditions: 50°C for 2min, 95°C for 10min, 40 rounds of 95°C for 15sec and 60°C for 1min. A dissociation cycle was performed after each run to check for non-specific amplification or contamination. The relative gene expression was estimated utilizing the 2ΔΔCt formula. A normalization value was generated through the Genorm program using GAPDH, HMBS and HPRT as housekeeping genes [20,21]. The set of primers, obtained using Primer Express (Applied Biosystems) was validated through BLAST and BLAT (sequences in Table 1).

**Table 1:** Primer sequences used for qRT-PCT. The sequences were obtained using Primer Express (Applied Biosystems) and validated through BLAST and BLAT.

Gene	Forward Primer 5`→ 3	Reverse Primer 5`→ 3
**Beta 1 Integrin**	CACGGATGCTGGGTTTCAC	CCATCATTGGGTAAAACAATACCA
**Δn p63**	ACCCGGGCCCACACA	GCTGACTTGGCAGTGCTTGA
**Alpha 6 Integrin**	TGTTACCCTTTGGCCTTCTTTC	TGCATTTGGCGTAGCAA
**Involucrin**	AAGGGCTTTCCCAAACATGA	TTTTGATCAGGCAGATCCTTCA
**Fibronectin**	CCGCTGGATGCGTTCAAC	GTGGTAGCCTCCGAACAGATG
**Nestin**	GGTCACTGTCGCCGCTACTC	AAGCGGACGTGGAGCACTA
**Vimentin**	GAGAGAGGAAGCCGAAGCA	GCCAGAGAAGCATTGTCAACATC
**GFAP**	CCAGCTTCGAGCCAAGGA	GAAGCTCCGCCTGGTAGACA
**Beta III Tubulin**	TCACGCAGCAGATGTTCGAT	GTGGCGCGGGTCACA
**CNPase**	AAGACAGCGTGGCGACTAGAC	CAGAGCTGCCATTGGTTCTTC
**Leptin**	TGTGCTGCAGATAGCCAATGA	TGGAGAAGGCCAGCAGATG
**Alkaline Phosphatase**	GAGTGAGCGCAGCCACAGA	TGTGACCTCATTGCCCTGAGT
**Collagen II**	GTGTGTGTGACACTGGGAATGTC	GGTTGAGGCAGTCTGGGTCTT
**GAPDH**	CATGGCCTTCCGTGTTCCTA	GCGGCACGTCAGATCCA
**HPRT**	TGAAAGACTTGCTCGAGATGTCA	CACACAGAGGGCCACAATGT

### Western Blotting

The lysates were obtained on ice using RIPA+ buffer supplemented with inhibitors of phosphatases and proteases (Amersham Biosciences, USA). Protein was quantified using the Bradford reagent (BioRad, USA). Equal amounts (50–100 μg) of protein from each culture were analyzed on a 7.5% or 10% polyacrylamide gel. After transfer to a nitrocellulose membrane and blocking, the primary and secondary antibodies were incubated and the signal was revealed using the ECL plus reagent (GE Healthcare, USA), as recommended by the manufacturer.

### Flow Cytometry

For analysis of the surface markers, the cell culture plates were placed in crushed ice and detached to single cell suspensions using ice-cold 50mM EDTA (Versene) solution. The cells were counted and then washed with phosphate-buffered saline (PBS). After blocking for 1h in 5% bovine serum albumin, a total of 10^6^ cells were incubated with specific antibodies for 30min at room temperature. Unbound antibody was washed out through three cycles of 10min washing with PBS and cells were analyzed on a BD FACSAria I/II flow cytometer (BD Biosciences, San Jose, CA, USA). The data were analyzed using FlowJo 7.6 and at least 50,000 events per sample were collected.

### Antibodies

The primary antibodies used were: anti-filaggrin (1:500; Abcam, catalog number # ab24584), anti-fibronectin (1:200; R&D Systems, catalog number # AF19181), anti-GFAP (1:400; R&D Systems, catalog number # AF2594), anti-βIII tubulin (1:200; R&D Systems, catalog number # MAB1195), anti-vimentin (1:200; R&D Systems, catalog number # MAB2105), anti-nestin (1:200; R&D Systems, catalog number # MAB2736), anti-CNPase (1:400; Chemicon, catalog number # MAB326), CD34-FITC (Pharmingen, San Jose, CA catalog number # 553733) and anti-p63 (1:500, AbCAM, catalog number # ab53039). The secondary antibodies were: Texas Red anti-sheep (1:1,000 catalog number # TI-6000), FITC anti-sheep (1:500 catalog number # F7634), FITC anti-rabbit (1:1,000 catalog number # FI-1000), anti-sheep HRP (1:1,000 catalog number # A16-147), anti-rabbit HRP (1:1,000 catalog number # PI-1000) all from Vector Laboratories, USA. The fluorophore-conjugated antibodies for flow cytometry were: CD34-PE catalog number # 551387, CD31-APC catalog number #561814, CD45-PE catalog number # 553081, CD44-PE catalog number # 561860, CD90-FITC catalog number # 554894, CD29-PE catalog number #562801, CD105-PE catalog number # 562759 diluted 1:500, all from BD Biosciences (San Jose, CA, USA).

### Statistical analysis

The data shown are the means of at least three independent experiments carried out in triplicates ±SEM. Statistical comparison was carried out by analysis of variance (one way ANOVA) with Tukey test for post hoc comparison using the GraphPad Prism 4 program. Two levels of confidence were employed: p < 0.05 (*) and p <0.001 (**). All analyses used as reference the first group shown in each graph.

## Results

### Epidermal Stem Cells may be selected from total epidermis by their ability to strongly adhere to type IV collagen coated dishes

**Fig 1:**
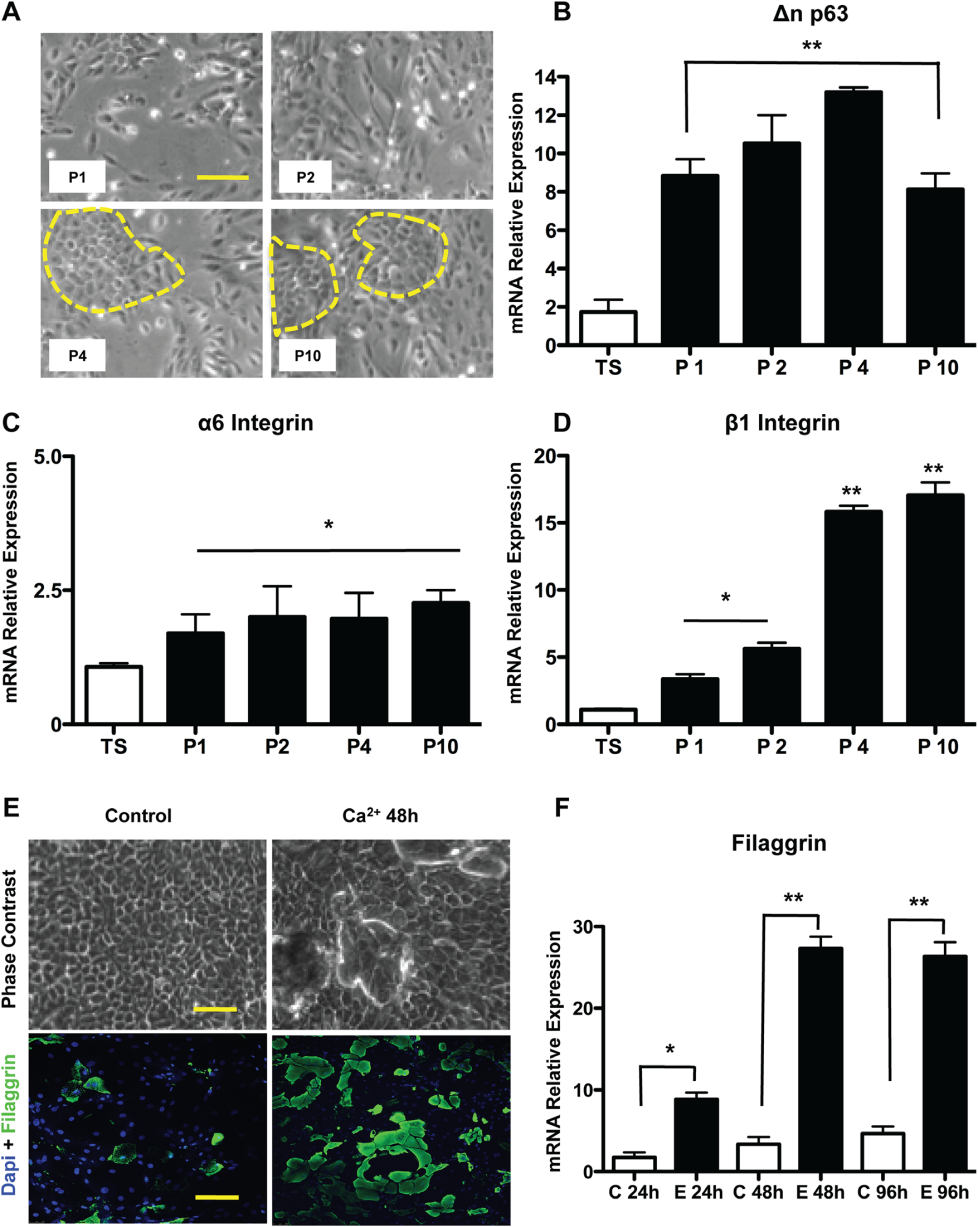
Isolation, characterization and differentiation of murine epidermal stem cells. The epidermal stem cells were simultaneously isolated with two other cell populations and selected for their ability to adhere to collagen IV. (A) Micrograph displaying holoclonic colonies at the 4^th^ through the 10^th^ passage of subculturing. mRNA relative levels for specific markers of these stem cells were evaluated by qRT-PCR: (B) Δn p63; (C) α6 integrin; (D) β1 integrin. The normalization factor was calculated with the GeNorm algorithm (HPRT, GAPDH and HMBS were used as endogenous control). (E) Cells after reaching the fourth passage were induced to differentiate by increasing levels of calcium/serum. The efficiency of this process was assessed by evaluating the mRNA levels (F) of a differentiation marker, namely filaggrin. The results are presented as the mean ± SD of values obtained in three independent experiments performed in triplicates. Statistical analyzes were performed using ANOVA analysis of variance followed by Tukey test. All groups were measured versus the control (TS = total skin, P1, P2, P4, P10 = passages from 1 to 10, C = control not induced to differentiate, E = Epidermal differentiation), ns = not significant; * p ≤ 0.05; ** p≤0.001.

Epidermal stem cells maintained in SMEM in the presence of 0.05mM Ca^2+^, 3% fetal bovine serum, strongly adhered to collagen IV coated plates. After the first plating at a density of 10^6^ cells /35mm plate, cultures were trypsinized for a short period of time so as to eliminate poorly adhered contaminant cells, leaving behind the EpiSCs adhered to the plate. After this procedure, uniform clusters of EpiSCs (20±7 clusters/plate) were grown for a week or two. At 80% confluency, cells were sub-cultured and named as passage 1 cells (P1). This procedure was repeated until the 10^th^ passage. Cells maintained a uniform morphology (Fig 1A) during the successive sub-culturing, with no signs of differentiation being observed.

### During consecutive passaging, the pool of EpiSCs Beta 1 Integrin and p63 cells, but not of alpha 6 Integrin cells, is upregulated

Passages 1, 2, 4 and 10 were chosen for RNA, protein and cell analysis. All comparisons were carried out using total skin (TS) as the reference. Analysis of the mRNA content by RT-real time PCR showed a significant increase in p63, since the first passage relative to total skin (Fig 1B, 8 fold, p<0.05), reaching the greatest level at the 4^th^ passage (12.5 fold, p<0.05), with a small decline to the 10^th^ passage. These data were validated by flow cytometry, with the relative proportions of p63+ cells ranging from 84 ± 6% to 92 ± 4% in the 4^th^ and 10^th^ passages respectively. The mRNA levels of alpha 6 integrin did not show any type of regulation as a function of sub-culturing (Fig 1C). Another epidermal stem cell marker, the beta 1 Integrin, was slightly increased in the first passage (Fig 1D, 3.36 fold, p<0.05) and in the 2^nd^ and 4^th^ passages (5.6 and 15.8 fold, respectively, p<0.05) and this expression remained stable at the 10th passage (17 fold, p<0.05). These data suggest that there was enrichment for EpiSCs during the first four passages, which was maintained up to the 10^th^ passage.

### Increased Fetal bovine serum concentration leads to EpiSC differentiation

Differentiation of EpiSCs into terminally differentiated keratinocytes was achieved through exposure to 10% FBS (what leads to increased Ca^2+^ levels) and removal of the type IV collagen coating. During the following 24 to 96h, morphological changes, such as elongation and compactation of the cells, could be observed. At 48h of differentiation, cell sobreposition could be clearly noted (Fig 1E), also, the cells presented increased levels of Filaggrin, indicating that the *in vitro* differentiation recapitulates the process that occurs *in vivo*. At 96h, the cells detached from the plate, remaining only a few clusters with altered morphology, and the levels of intermediate filaments typical of terminally differentiated keratinocytes, such as Filaggrin (Fig 1F), increased by 27.3 and 26.3 fold at, respectively at 48 and 96h (p<0.001).

### Enrichment for SKPs, as demonstrated by Nestin, Fibronectin and Vimentin upregulation

**Fig 2:**
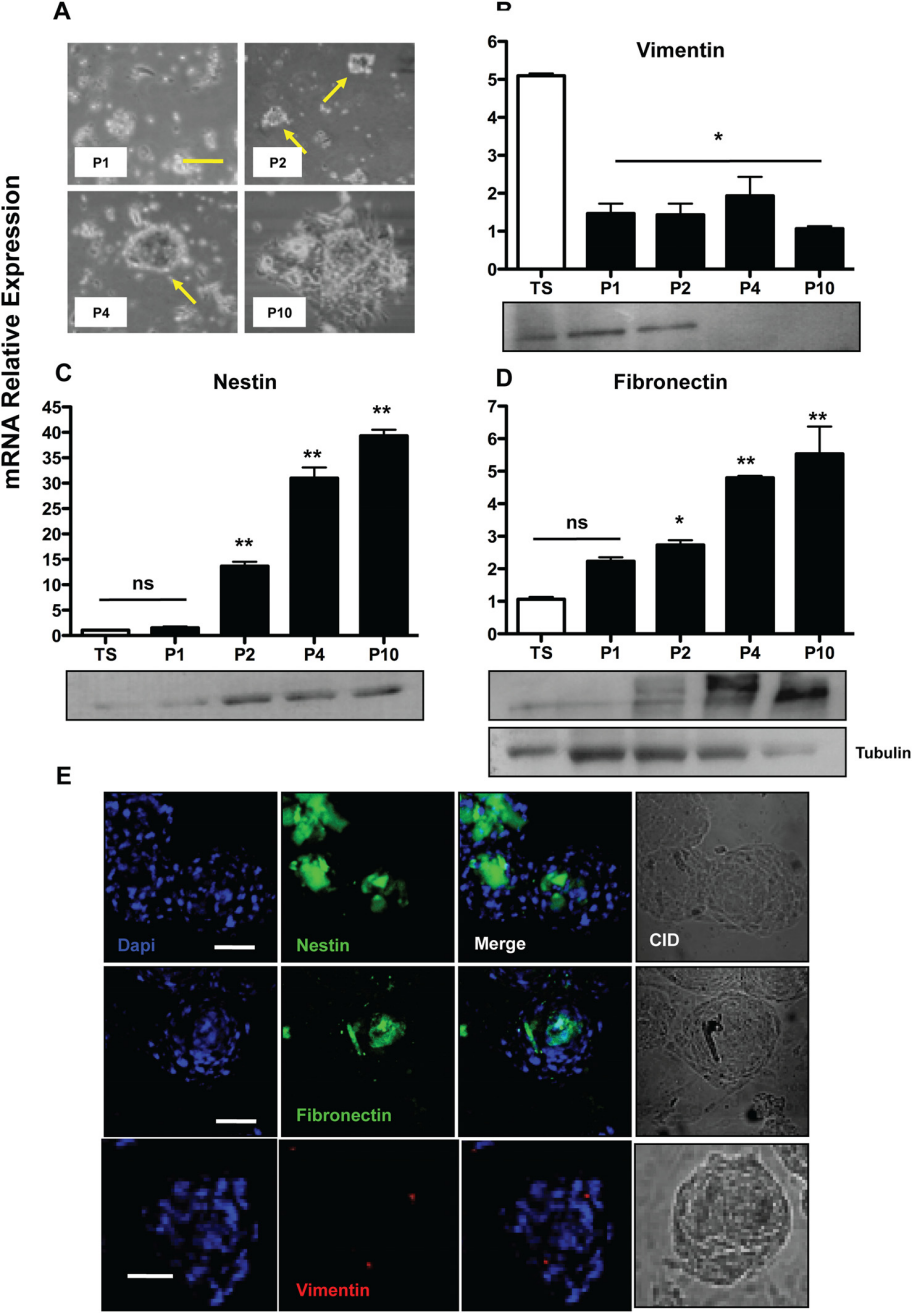
Isolation and characterization of skin stem cell precursors (SKP). The SKPs were selected by culture in serum-free suspension, generating cell aggregates, as may be observed in the micrograph shown in (A). To assess the success of selection, we analyzed the levels of mRNA and protein of negative (B) Vimentin and positive (C) Nestin and (D) Fibronectin markers through qRT-PCR and Western blotting. The same markers were observed with fluorescence microscopy to show the distribution/localization of stem cells in the mass of aggregates (E) Bar = 50μm. The results are presented as the mean ± SD of values obtained in three independent experiments performed in triplicates. Statistical analyzes were performed using ANOVA analysis of variance followed by Tukey test to post-Kramer. All groups were assessed versus control (in the case of stem cell markers relative to the total dermis, TS = total skin), ns = not significant; * p ≤ 0.05; ** p≤0.001.

**Fig 3:**
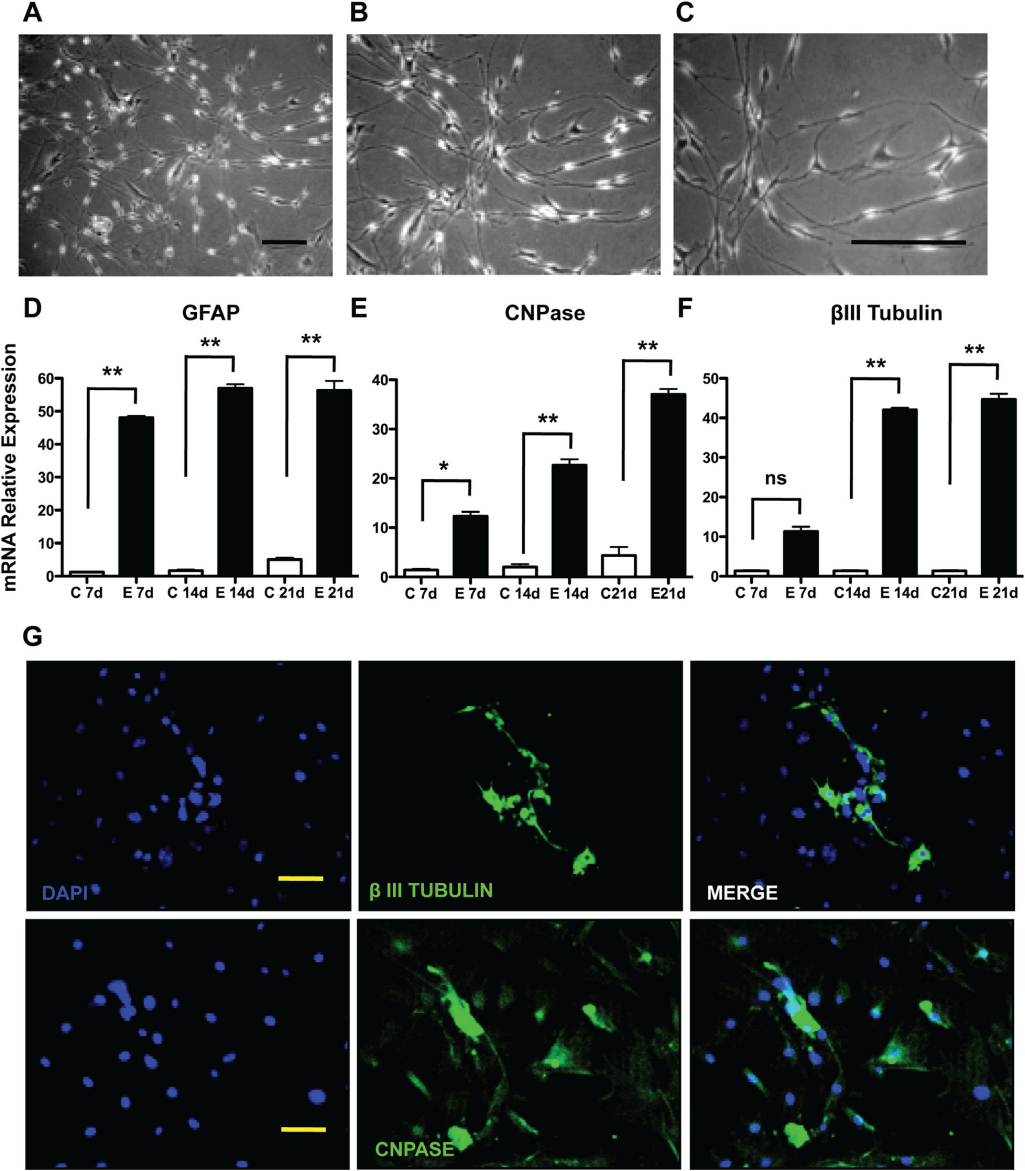
Differentiation of murine skin stem cell precursors (SKP). The differentiation of SKPs was induced through the dissociation of cell aggregates into single cells followed by serum exposure and, as may be seen in the micrographs after 7 days (A), 14 days (B) and 21 days (C), these cells presented several elongated extensions. The mRNA levels of astrocyte markers (D) GFAP; oligodendrocytes (E) CNPase; and neurons (F) βIII tubulin were measured by qRT-PCR and validated at the protein level (G) by immunofluorescence microscopy, Bar = 50μm. The results are presented as the mean ± SD of values obtained in three independent experiments performed in triplicates. Statistical analyzes were performed using ANOVA analysis of variance followed by Tukey test to post-Kramer. All groups were measured versus the undifferentiated control in the shortest time of differentiation, ns = not significant; * p ≤ 0.05; ** p≤0.001.

SKP floating spheres became evident at the 2nd passage (around 20 ± 3 colonies on average for 10^6^ cells plated), becoming more prominent at the 4^th^ passage, (Fig 2A). They could be identified as uniform floating cell clusters, which, upon plating onto adherent plates, were capable of adhering and proliferating, acquiring a fibroblastoid phenotype (Fig 2A). The cells which were incapable of growing in suspension ended up adhering to the surface after consecutive passaging, leading to enrichment for SKPs, as suggested by increased mRNA content of positive SKPs markers, such as Fibronectin and Nestin. The negative marker, Vimentin, expressed in mesodermal lineages, showed a decrease after the first passage (Fig 2B, 1.46 fold, p<0.001), but, apparently, no further negative selection of the mesodermal cells occurred in the following sub-culturing, since the fold change did not decrease. The opposite behaviour was observed for the levels of Nestin, a SKP positive marker, with increases of 13.6 fold in the 2nd passage (Fig 2C, p<0.001) and even greater values for the 4^th^ and 10^th^ passages (31 and 39.3 fold, respectively p<0.001 Fig 3F). As for Fibronectin, a significantly increased mRNA content was observed at the 4^th^ (Fig 2D, 4.7 fold) and 10^th^ (5.5 fold) passage (p<0.001, Fig 3E). These data were confirmed at the protein level by Western blotting. The same results were obtained by immuno-localization of these three markers (Fig 2E). Nestin and Fibronectin were more expressed in the inner core of the spheres, whereas Vimentin showed an irregular distribution throughout the spheres, apparently marking a few cells at the clusters periphery.

### SKPs differentiate into putative βIII tubulin^+^, CNPase^+^ and GFAP^+^ neural precursors.

SKP spheres were enzymatically dissociated and plated onto poly-L-lysine coated plates in DMEM:F12 at a 3:1 ratio, supplemented with 15% FBS, used as a neurogenesis induction medium. After 14 days, alterations in morphology could be observed (Fig 3B), since the cells became thinner and emitted long cytoplasmatic extensions. Neural precursors differentiation was detected through GFAP assessment. The mRNA level for this protein increased 47 fold at day 7, followed by a greater fold change on the 14th and 21th days (56 fold and 55 fold, respectively), as shown in Fig 3D (p<0.001). These cells also showed increased CNPase mRNA expression (Fig 3E) and immuno-localization (Fig 3G). Since day seven, the mRNA expression for Beta III tubulin increased (Fig 3F, 36 fold, p<0.001), and these levels were maintained high at the 14th and 21th day (42 and 44 fold, respectively, p<0.001), as shown in Fig 3G. The localization of these markers was confirmed by confocal microscopy (Fig 3G).

### Mesenchymal stromal dermis-derived stem cells are CD34^-^CD31^-^CD45^-^CD44^+^CD90^+^CD29^+^CD105^+^

**Fig 4:**
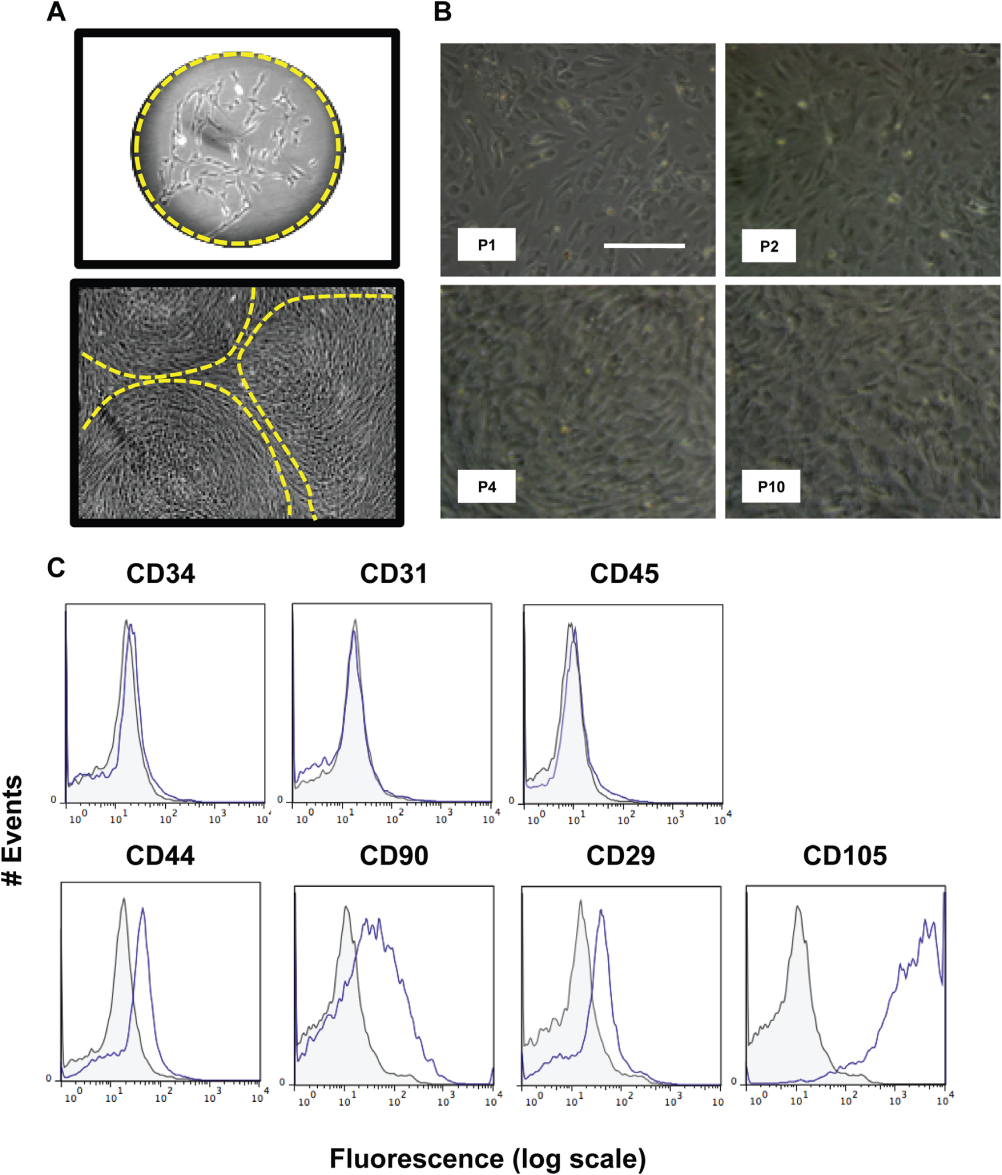
Isolation and characterization of mesenchymal stromal stem cells from murine dermis. The mesenchymal stem cells from murine dermis were selected for their ability to form colonies at low densities (CFU), as shown in the micrograph (A). These colonies expand and form a confluent layer at P0, as shown in (A). These cells are then passaged whenever they reached 80% confluence and expanded until passage 10 (B). Cells at P4 were used to detect the presence of positive and negative markers of MSCs by flow cytometry, namely: CD34, CD31, CD45, CD44, CD90, CD29 and CD105 (C). All groups marked with conjugated antibody (blue line) were compared to their respective controls (gray filled line); at least 50.000 events were collected for analysis. IgG isotype controls conjugated with Alexa 488 and APC were used as negative controls. Results are representative of those obtained in three independent experiments performed in triplicates.

Mesenchymal stem cells were maintained in DME supplemented with 10% FBS. After 1 to 2 weeks, these cells formed isolated colonies (Fig 4A), which grew, being sub-cultured before cells reached 80% confluency. These cells were kept in culture up to the 10^th^ passage. Photomicrographs taken at P1 through P10 (Fig 4B) demonstrated that upon *in vitro* maintenance, no morphological changes occurred. At the 4^th^ passage, we performed flow cytometry analysis of several clusters of differentiation expression markers (Fig 4C for representative histograms). Consistently presented less than 5% of the population of these cells were positive for the following markers: CD34, 31 and 45 (p<0.001). They also showed a high percentage of CD44^+^ cells (65%±7, p<0.001), as well as CD90^+^ (82%±10, p<0.05), CD29^+^ (72%±4, p<0.001) and CD105^+^ (92%±5, p<0.001).

### Mesenchymal stem cells differentiate into osteocytes, adipocytes and chondrocytes

**Fig 5:**
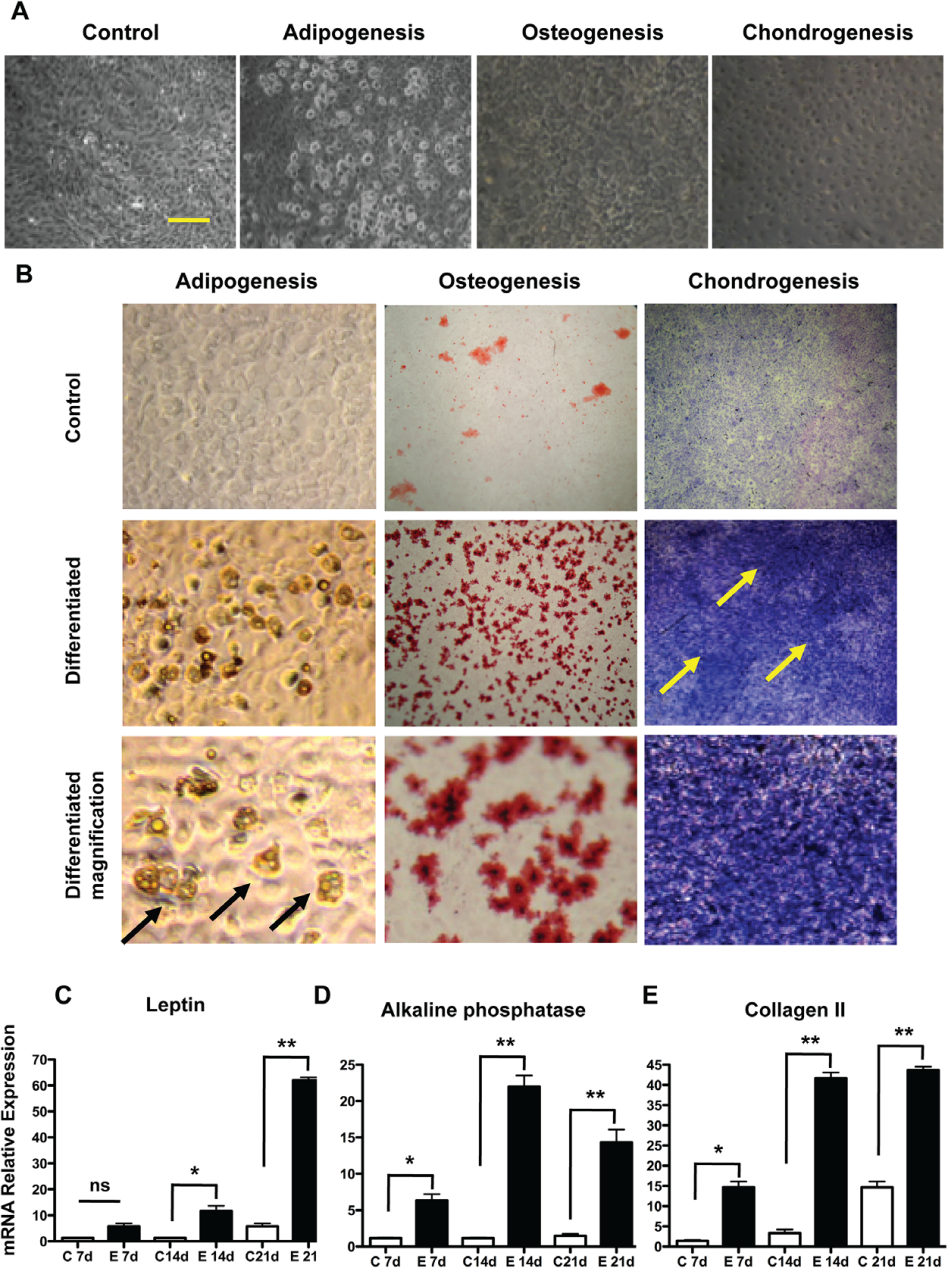
Mesenchymal stem cells were stimulated to undergo adipogenesis, osteogenesis and chondrogenesis. Cells were exposed to differentiation medium at P4 and samples were collected at 7, 14 and 21 days. Important morphological changes may be seen in micrographs under an inverted microscope after 21 days (A), Bar = 50μm. The same is reflected in increased production of lipid droplets (adipogenesis), calcified matrix (osteogenesis) and glycosaminoglycans (chondrogenesis) as shown by immune-histochemistry (B), the control group is in the inserts (21 days after induction). The relative expression levels of mRNA for specific markers of these processes were evaluated in (C) Leptin; (D) Alkaline Phosphatase; (E) Collagen II. The results are presented as the mean ± SD of values obtained in three independent experiments performed in triplicate. Statistical analyzes were performed using ANOVA analysis of variance followed by Tukey test. All groups were measured *versus* the undifferentiated control in the shortest differentiation period of time, ns = not significant; * p ≤ 0.05; ** p≤0.001.

Mesenchymal skin stem cells were capable of adipogenesis, with increased number of lipid storing cells being more appreciable at the 21^st^ day of induction (Fig 5A), but the leptin mRNA content was increased since the 14^th^ day (Fig 5C, 12 fold, p<0.001), and on the 21^st^ day the fold change was superior to 60 (p<0.001). Oil red O positive cells were the majority in the population at the 21^st^ day of differentiation (Fig 5B). Osteogenic differentiation was achieved from day 14, as indicated by the Alizarin Red staining (Fig 5B) and, also, by increased alkaline phosphatase mRNA content (more than 20 fold at 14^th^ day, as shown in Fig 5D, (p<0.001) decreasing to 15 fold at the 21^st^ day. Chondrogenesis became evident at the 14^th^ day, when the expression level for collagen II, a specific chondrocytes marker, reached changes superior to 40 fold (p<0.001), as shown in Fig 5E, a result which was confirmed by microscopy and Toluidin blue staining (Fig 5A and 5B).

## Discussion

The skin has long been recognized as having resident adult stem cell populations, which allow for the equilibrium between cell proliferation and differentiation and support this highly dynamic tissue. To replace cells of these various lineages, compartments of these stem cells are located in several niches, including the interfollicular epidermis, sebaceous glands, dermis and hair follicles [22]. Herein, we describe a simple method of simultaneous isolation of three different subsets of skin stem cells, using a murine model. Since our main objective was to develop a robust and simple protocol, we have chosen to use multistep enzymatic digestions that have been proven as suitable for other models [23,24]. After collagenase and dispase treatment, we have incubated the resulting cells originated from the epidermal tissue under the same culture conditions taken as optimal for the growth of these cells [1,25]. This led to the growth of lineages with epidermal stem cell morphology, which were capable of expansion *in vitro* up to the 10^th^ passage. Aiming at characterizing the real identity of these cells, we examined beta 1-integrin mRNA levels, and found a significant increase of this positive marker upon sub-culturing. It had already been described in the literature that this protein is highly expressed in EpiSCs [1]. The same pattern of up-regulation could not be seen for the expression of α6 integrin, since the mRNA levels for this transmembrane protein were not significantly altered. Whether expression of α6 integrin (in combination with low expression of CD71) identifies murine epidermal stem cells, has been the object of dispute among different groups [26], but, apparently, in our model, this marker does not seem to be significantly up-regulated. To further characterize these cells, we analyzed the expression of p63, a transcription factor which is likely to play a key role in regulating self-renewal and the long-term proliferative capacity of epidermal stem cells [27]. The ΔN isoform of p63, a member of the p53 family of proto-oncogenes, is preferentially expressed in basal epidermal cells, with its mRNA levels being increased during the sub-culturing steps. Having estabilished the expression of EpiSC markers, we addressed whether these cells could differentiate into terminally differentiated keratinocytes. After exposure to fetal calf serum, morphological changes and increased filaggrin expression could be observed. Interestingly, during *in vivo* cornification of the epidermis, this intermediate filament was also shown to be up regulated [8]. These results indicate that the cells obtained using our protocol were suggestively the same as those described by other groups [3,5,6,10,12–15] and that they were capable of terminal, or rather, partially terminal differentiation, into keratinocytes, since the processes are not resumed without atmospheric concentrations of oxygen not found in liquid culturing [28].

It has been known that adult mammalian dermis is also a source of multi-potent positive precursor cell populations, which are isolated using spheroid cultures (termed SKPs for skin-derived precursors), which are nestin-positive (a marker of neural progenitor cells) in culture and differentiate into neurons, glia, smooth muscle cells, and adipocytes [13–15]. One possibility is that these spheres would arise from melanocytes present in the albino strain Balb/c, but this was previously ruled out since these cells are not proficient to grow in the selected medium initially described by Fernandes and cols [13– 15]. We could observe that upon sub-culturing, cell spheres were formed, expressing high levels of fibronectin and nestin, and being negative for the mesodermal markers, such as vimentin. Vimentin protein levels, which are more reliable than the mRNA levels, were down-regulated during sub-culturing most probably by means of negative selection of the contaminant fibroblasts in the non-adherent populations. Moreover, through immune-localization, it was possible to observe that the highest levels of the positive markers were expressed in the inner core of the spheres, whereas contaminant mesodermal cells were sparse, displaying an irregular distribution in the spheres architecture. Upon induction, these cells were capable of differentiating into neural precursor lineages. This could be observed through increased mRNA levels at early differentiation periods of time and, later on, by immune-localization. Differentiation was achieved, as may be noted by the drastic morphological changes and CNPase, GFAP and βIII tubulin up-regulation. Therefore, it is possible to state that the cells retrieved using our method were comparable to those described in the literature [13–15]. The stromal mesenchymal stem cells obtained from the dermis were sub-cultured as recommended in the literature for other types of mesodermal-derived stem cells [29]. We also performed the full characterization of the expression of several cluster of differentiation surface markers in order to compare the retrieved cells with those from other organs obtained in the literature and found that the CD34^-^CD31^-^CD45^-^CD44^+^CD90^+^CD29^+^CD105^+^ expression profile, which is very similar to those found for bone marrow and adipose tissue [30]. MSCs have been shown to have considerable plasticity, being capable of differentiation into adipocytes, chondrocytes and osteocytes [31]. Upon specific induction, these mesenchymal stem cells were shown to be capable of undergoing adipogenesis, through accumulation of lipid storing vacuoles, which were stained by Oil Red O. Furthermore, these cells presented high levels of leptin mRNA during the course of differentiation. Osteogenesis was also addressed and achieved, being corroborated by Alizarin Red positive staining and increased levels of alkaline phosphatase mRNA, two standard methods for this type of differentiation. In addition, these cells also show a typical osteogenecic-associated phosphorylation pattern when inquired through phosphoproteomics [19]. Chondrogenesis was screened by Toluidine blue staining and collagen type II expression, with both parameters being positive in our model, suggesting that chondrocyte-like cells were obtained. Therefore, they displayed the most important markers and characteristics that a primary culture should posesses in order to be classified as a MSC following the recommendations of the International Society for Cell Therapy [29].

In brief, several described protocols attempted to isolate different stem cell populations from the skin, but none of them afforded simultaneous isolation of more than one population. Being capable of obtaining different adult stem cells from the same organ/tissue is a critical issue in attempts to develop future cell therapy clinical protocols. This enables that a wide range of differentiated cell types to be achieved, since the potentiality of adult stem cells is quite limited.

We were capable to develop a simple and practical method, based on enzymatic digestion and selective sub-culturing, which allows isolating different skin stem cells populations. When compared to other described protocols in the literature this represents significant advantage since it can retrieve not only one but three different stem cell populations from the same tissue sample.

One of the major advantages of the herein described technique is that it does not require expensive materials nor high-tech facilities, such as flow cytometer based cell sorters, to be employed with success. It is important to note that the method was developed using a murine skin model, but we are currently attempting to translate this protocol to the human skin aiming at developing cell-based therapy of degenerative diseases.

## Conclusion

Skin stem cells have been known for a long time, however, no current protocol is available to isolate more than one skin SC population at the same time. We have simultaneously isolated and characterized three stem cell populations of the dermis and epidermis from murine skin, utilizing a simple and practical protocol based on culture selection techniques. These cells are shown to be capable of generating chondrocytes, adipocytes, osteocytes, terminally differentiated keratinocytes, neurons and glia, rendering this protocol suitable for the isolation of cells to be used for tissue replenishment, cell-based therapies and regenerative medicine.

## Supporting Information

S1 Fig Overview of the protocol for simultaneous isolation of three different stem cell populations from epidermis and dermis from murine back skin. After euthanasia, the backsin was shaved to remove fur and the hypodermis. Liberase digestion for 1h at 37°C was sufficient to dissociate the cells from the dermis and the remaining epidermis was exposed to another cycle of digestion in the presence of dispase for 30min at 37°C and single epidermal cells were obtained as may be seen in the scheme depicted in **(A)**. The cells retrieved from the dermis were plated in selection media for enrichment of mesenchymal stem cells, which were later differentiated into adipo, osteo and chondrocytes **(B)** and for SKPs, which were later differentiated into neural precursors **(C)**. The epidermal compartment was seeded in selection media for epidermal stem cells, which were later differentiated into keratinocytes **(D)**.
(TIF)

S2 Fig Positive and negative controls for antibodies used for immunofluorescence. Dermis from mice was stained as a positive control for Fibronectin **(A)** and Vimentin **(B)** and the epidermis was used as negative control as seen for Fibronectin **(C)** and Vimentin **(D)**. Bar = 100μm.
(TIF)
