# Supplementary figures and images for: Simultaneous Isolation of Three Different Stem Cell Populations from Murine Skin
Source: PLoS One. 2015 Oct 13;10(10):e0140143. doi: 10.1371/journal.pone.0140143 (PMC4604199; doi:10.1371/journal.pone.0140143)

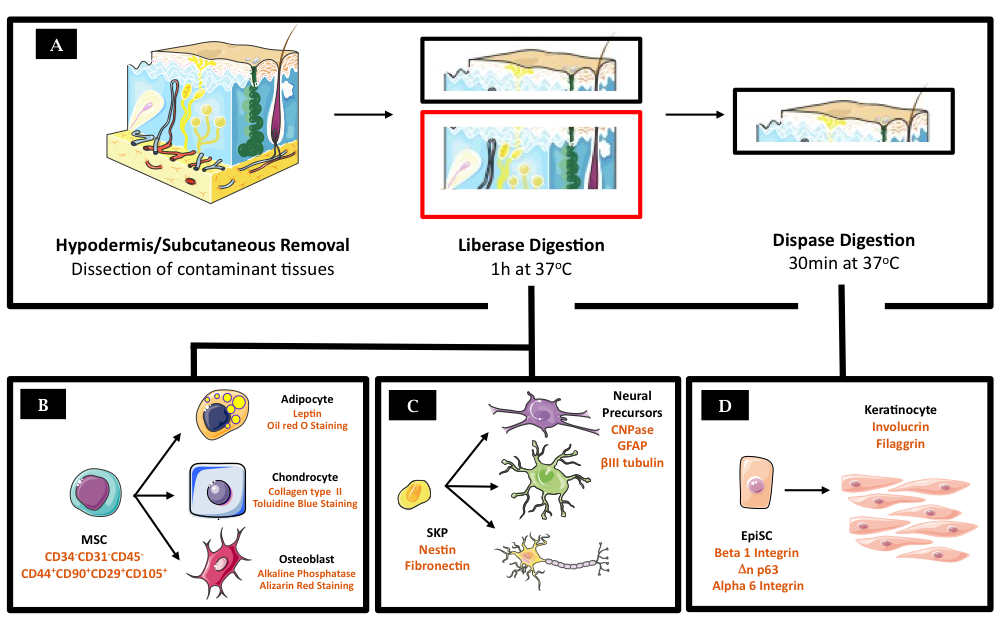

Supplement: S1 Fig — After euthanasia, the backsin was shaved to remove fur and the hypodermis. Liberase digestion for 1h at 37°C was sufficient to dissociate the cells from the dermis and the remaining epidermis was exposed to another cycle of digestion in the presence of dispase for 30min at 37°C and single epidermal cells were obtained as may be seen in the scheme depicted in (A). The cells retrieved from the dermis were plated in selection media for enrichment of mesenchymal stem cells, which were later differentiated into adipo, osteo and chondrocytes (B) and for SKPs, which were later differentiated into neural precursors (C). The epidermal compartment was seeded in selection media for epidermal stem cells, which were later differentiated into keratinocytes (D). (TIF) [file pone.0140143.s001.tif]
